# Effects of Aging on the Color and Translucency of Monolithic Translucent Y-TZP Ceramics: A Systematic Review and Meta-Analysis of *In Vitro* Studies

**DOI:** 10.1155/2021/8875023

**Published:** 2021-01-25

**Authors:** Chang-yuan Zhang, Check Agingu, James Kit Hon Tsoi, Hao Yu

**Affiliations:** ^1^Fujian Key Laboratory of Oral Diseases & Fujian Provincial Engineering Research Center of Oral Biomaterial & Stomatological Key Laboratory of Fujian College and University, School and Hospital of Stomatology, Fujian Medical University, Fuzhou 350002, China; ^2^Faculty of Dentistry, University of Hong Kong, Hong Kong SAR, China; ^3^Department of Applied Prosthodontics, Graduate School of Biomedical Sciences, Nagasaki University, Nagasaki, Japan

## Abstract

**Background:**

Monolithic restorations made of translucent yttria-stabilized tetragonal zirconia polycrystal (Y-TZP) have become popular over the past few decades. However, whether aging affects the color and translucency of monolithic translucent Y-TZP is unclear.

**Objective:**

The aim of this systematic review and meta-analysis of *in vitro* studies was to evaluate the effects of aging on the color and translucency of monolithic translucent Y-TZP ceramics.

**Materials and Methods:**

This systematic review/meta-analysis was reported according to the PRISMA statement and registered in the OSF registries (https://osf.io/5qjmu). Four databases including Medline via the PubMed, Embase, and Web of Science databases and the Cochrane Library were searched using no publication year and language limits. The last search was executed on November 20, 2020. *In vitro* studies comparing the translucency and/or color of monolithic translucent Y-TZP ceramics before and after simulated aging were selected. Meta-analyses were performed using Review Manager software (version 5.3, Cochrane Collaboration, Oxford, UK) with random-effects models at a significance level of 0.05. A risk-of-bias assessment was also performed for the included studies.

**Results:**

Of the 188 potentially relevant studies, 13 were included in the systematic review. The hydrothermal aging duration ranged from 1 to 100 h at relatively similar temperatures (~134°C). In the general meta-analyses, the aged Y-TZP ceramics exhibited similar translucency parameter (TP), L^∗^, and b^∗^ values compared with the nonaged controls (*P* = .73, *P* = .49, and *P* = .62, respectively). Moreover, there was a significant difference between the aged and nonaged Y-TZP ceramics in the a^∗^ value (*P* = .03; MD = −0.26; 95% CI = −0.51 to − 0.02), favoring the nonaged Y-TZP ceramics. The subgroup analyses showed that the duration of aging contributed to changes in the translucency and color of the Y-TZP ceramics.

**Conclusions:**

The optical properties of monolithic translucent Y-TZP ceramics were stable after hydrothermal aging at 134°C and 0.2 MPa for ≤20 h. Moreover, clinically unacceptable changes in the translucency and color of monolithic translucent Y-TZP ceramics were found after hydrothermal aging for >20 h.

## 1. Introduction

The popularity of dental zirconia has increased in recent decades because of its excellent mechanical characteristics, biocompatibility, and acceptable esthetic properties [1, 2]. A questionnaire-based survey regarding the selection of dental ceramic materials reported that dental zirconia was one of the top choices for both anterior (layered) and posterior (monolithic) restorations [3].

At ambient pressure, zirconia can exhibit 3 allotropic crystal phase structures: a monoclinic phase (*m*) from room temperature to 1170°C, a tetragonal phase (*t*) from 1170°C to 2370°C, and a cubic phase (*c*) above 2370°C to its melting point at 2715°C and boiling point of 4300°C [4, 5]. To stabilize the *t* and *c* phases of zirconia at room temperature, the addition of different amount of stabilizing oxides, such as yttria (Y_2_O_3_), to pure zirconia crystals is essential and well studied [2, 4, 6]. In particular, *t* phase zirconia is useful in dentistry because of its strength [2, 6]. Therefore, yttria-stabilized zirconia polycrystal (Y-TZP) has been widely used as a framework for fixed dental prostheses (FDPs) and monolithic restorations [7]. To date, there are three generations of Y-TZP ceramics (1^st^, 2^nd^, and 3^rd^ generations) in dentistry [2]. First-generation Y-TZP ceramics are 3 mol% (5.2 wt%) Y-TZP (3Y-TZP) containing 0.25 wt% alumina, which are highly opaque. Second-generation Y-TZP ceramics are refined by reducing the concentration of alumina (<0.05 wt%) and sintering at a higher temperature (~1450°C) of 3Y-TZP [2]. To further reduce opacity, 3^rd^ generation Y-TZP ceramics are refined by increasing the yttria content to 4 and 5 mol% (4Y-TZP and 5Y-TZP) to stabilize the *c* phase content (>25%) [2]. Both 2^nd^ and 3^rd^ generations of Y-TZP ceramics are considered translucent and are indicated for posterior and/or anterior monolithic crowns and FDPs [2, 8].

Although *c* phase zirconia does not undergo stress-induced transformation [8], the 2^nd^ and 3^rd^ generations Y-TZP still have *t* phase so that *t-*to-*m* phase transformation will eventually be activated and accelerated when the Y-TZP ceramic is subjected to a humid environment with constant temperature changes, which is usually referred to as aging or low-temperature degradation (LTD) [9–13]. Evidence of aging has been observed in zirconia used in hip implants [14]. In fact, the mechanism of aging has been described using different theories and speculations [15, 16]. For example, water vapor has been proposed to attack the Zr–O bond and be incorporated into zirconia grains by filling oxygen vacancies; then, aging proceeds into the bulk material and jeopardizes the molecular and mechanical properties of Y-TZP ceramics [17, 18]. On the other hand, Lange et al. [19] proposed that water reacts with Y_2_O_3_ to form clusters rich in Y(OH)_3_, which leads to the depletion of the stabilizer in the surrounding zirconia grains and induces aging. This mechanism has also been supported by a recent study [20].

Despite the fact that various aging mechanisms have been proposed, the effects of aging on Y-TZP ceramics are still being studied and reported in the literature [13, 21–23]. The simulated aging of Y-TZP ceramics has commonly been performed by steam autoclave at 120°C to 140°C [16, 24–27]. A recent systematic review concluded that hydrothermal aging promoted LTD, as shown by the *t-*to-*m* phase transformation, and it negatively influenced the flexural strength of Y-TZP ceramics [18]. Moreover, the influences of aging on the surface roughness, surface microhardness, and fracture toughness of Y-TZP ceramics have been previously reported [9, 28–33].

Apart from mechanical properties, optical properties, including color and translucency, are critical for the long-term success of ceramic restorations, especially monolithic restorations [27, 34–36]. However, very limited information concerning the effects of aging on the optical properties of monolithic translucent Y-TZP ceramics (2^nd^ and 3^rd^ generations) is available. Han et al. [6] reported that autoclaving Y-TZP did not change its color, whereas other treatments such as ultraviolet and gamma irradiation changed the color of Y-TZP. Rafael et al. [37] reported significant differences in the lightness, chroma, and hue of Y-TZP ceramics in all groups after aging. In contrast, other studies have reported that Y-TZP ceramics can be considered color stable after a stimulated aging process [38, 39]. In addition to the color, efforts have also been made to investigate the effects of aging on the translucency of monolithic translucent Y-TZP ceramics. Current studies in the literature have shown that the translucency of Y-TZP ceramics is reduced [25, 40] or remains unchanged [41] after aging.

Theoretically, Y-TZP ceramic aging may lead to increased surface roughness and pigment breakdown, jeopardizing the esthetic outcome and stability of ceramic restorations [25]. The effects of aging on the color and translucency of monolithic zirconia were reviewed by Papageorgiou-Kyrana et al. [42]. However, no systematic review or meta-analysis has been performed in this field. Therefore, this systematic review and meta-analysis aimed to evaluate the effects of aging on the translucency and color of monolithic translucent Y-TZP ceramics.

## 2. Material and Methods

This systematic review and meta-analysis was performed according to the Preferred Reporting Items for Systematic Reviews and Meta-Analyses (PRISMA) statement [43] and registered in the OSF registries (https://osf.io/5qjmu). A systematic electronic literature search was conducted in Medline via PubMed, Embase, Web of Science (ISI—Web of Knowledge), and Cochrane Library with no publication year and language limits. The search terms and their combinations used in the literature search are listed in Supplemental Table 1. The last search was executed on November 20, 2020. The PICO questions were defined as follows: P: population—monolithic translucent Y-TZP ceramics; I: intervention—Y-TZP ceramics subjected to aging; C: control—Y-TZP ceramics not subjected to aging; O: outcome—an evaluation of color and translucency changes of Y-TZP ceramics; and S: study designs—in vitro studies. The primary evaluated outcome was the translucency of monolithic translucent zirconia, and the secondary evaluated outcome was the color of monolithic translucent zirconia.

Articles that met the following inclusion criteria were included: (1) studies that evaluated the effect of aging on the translucency and/or color of monolithic translucent Y-TZP ceramics and (2) studies that used translucency and/or color measurements according to ISO/TR 28642:2016 [44]. Articles meeting one or more of the following criteria were excluded: (1) study materials other than monolithic translucent Y-TZP ceramics; (2) reviews, protocols, clinical guidelines, and editorial letters; and (3) studies not using hydrothermal aging [33]. Two reviewers (C.Z. and A.C.) independently performed the literature searches and the study selection. Any disagreements were resolved by discussion or by consultation with another reviewer (H.Y.) [33]. The reference lists of all the selected articles were manually reviewed, and the full texts of potentially related studies were examined [45]. Lastly, manual searches were conducted in the following principal periodicals specific to the area of study: Journal of Prosthetic Dentistry, Journal of Dental Research, Journal of Dentistry, Operative Dentistry, Clinical Oral Investigations, Journal of Oral Rehabilitation, International Journal of Prosthodontics, Journal of Prosthodontic Research, Dental Materials Journal, and Journal of Prosthodontics.

A protocol for data extraction was defined and evaluated by 2 reviewers (C.Z. and A.C.) [33]. The following data were extracted from the included studies: demographic information (e.g., authors, publication year, and publication journal and title), the materials tested, the aging protocol, the mean and standard deviation of translucency and/or color, the sample size, and the evaluation methods.

The risk-of-bias assessment was based on a protocol adapted from previous systematic reviews [46, 47]. Briefly, the following parameters were used for the quality assessment: clearly specified aging protocol, sample size calculation, specimen randomization, adequate statistical analysis, ceramic sintering followed the manufacturers' instructions, and tests executed by a single-blinded operator [33]. If a parameter is reported, the study received a “Y”; if the information was missing, the study received an “N.” Studies that included 1 or 2 “Y” items were classified as having a high risk of bias, 3 or 4 “Y” items as a medium risk of bias, and 5 to 6 “Y” items as a low risk of bias [33].

For the meta-analysis, studies that did not present data on the translucency parameter (TP) and/or Commission Internationale de L'Éclairage (CIE) L^∗^, a^∗^, and b^∗^ values were excluded. Studies containing the color difference, contrast ratio, and percentage of total transmittance of light were not considered because of insufficient data. For studies that evaluated more than 1 type of ceramic material or 1 aging duration, all the relevant experimental (aged) groups were combined into a single group, and all the relevant control groups were combined into a single control group according to the Cochrane Statistical Guidelines [48]. All analyses were conducted using Review Manager software (version 5.3; Cochrane Collaboration, Oxford, UK) by employing a random-effects model at a significance level of 0.05. The mean difference (MD) and 95% confidence interval (CI) were calculated for the included studies. Subgroup analyses were performed to explore the potential causes of heterogeneity, including the type of monolithic translucent Y-TZP material (3Y-TZP vs. 5Y-TZP) and the steam autoclave duration (≤20 h vs. >20 h). For the studies included in the subgroup analyses, all the relevant groups were combined into a single subgroup (e.g., 3Y-TZP or 5Y-TZP for the material type) within a given study [48].

## 3. Results

**Figure 1:**
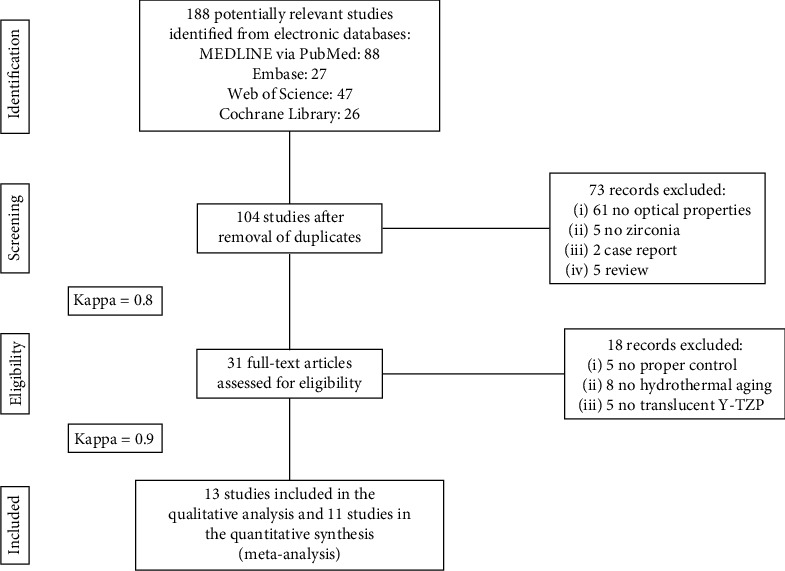
Flow diagram of study selection according to the PRISMA statement. PRISMA: Preferred Reporting Items for Systematic Reviews and Meta-Analyses.

Thirteen studies were included in the systematic review, and 11 studies were included in the meta-analysis (Figure 1). The characteristics of the included studies are presented in Table 1. The majority of the included studies (9 studies) presented a medium risk of bias, while 2 studies presented a high risk of bias and 2 presented a low risk of bias (Table 2).

The included articles were all in English and were published between 2014 and 2020. Of the 13 studies included in the systematic review, 1 study performed color measurements [37], 8 studies performed translucency evaluations [13, 39–41, 49–52], and 4 studies performed both types of investigations [22, 25, 53, 54]. All included studies were laboratory studies measuring the color and/or translucency with a spectrophotometer. All studies included in the meta-analysis adopted hydrothermal aging according to the ISO 13356:2015 [55]. The simulated aging time ranged from 1 to 100 h at relatively similar temperatures (~134°C).

**Figure 2:**
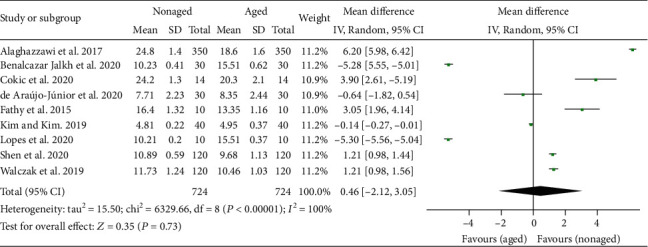
Forest plot summarizing the TP values of aged and nonaged Y-TZP ceramics. CI: confidence interval; SD: standard deviation.

The results of the general meta-analysis on translucency (Figure 2) showed no significant difference in the TP value between the nonaged and aged Y-TZP (*P* = .73; mean difference (MD) = 0.46; 95% confidence interval (CI) = −2.12 to 3.05).

**Figure 3:**
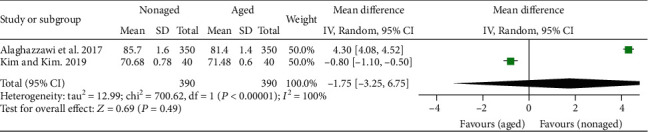
Forest plot summarizing the L^∗^ values of aged and nonaged Y-TZP ceramics. CI: confidence interval; SD: standard deviation.

**Figure 4:**
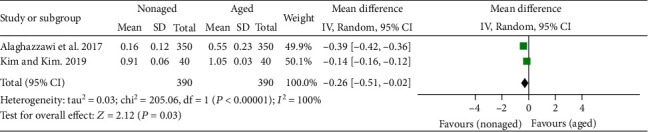
Forest plot summarizing the a^∗^ values of aged and nonaged Y-TZP ceramics. CI: confidence interval; SD: standard deviation.

**Figure 5:**
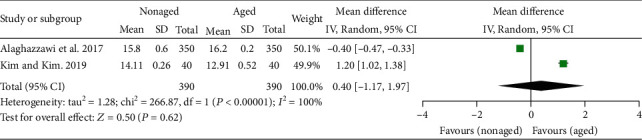
Forest plot summarizing the b^∗^ values of aged and nonaged Y-TZP ceramics. CI: confidence interval; SD: standard deviation.

The results of the general meta-analysis on the L^∗^ values showed no significant difference in the L^∗^ value between the nonaged and aged Y-TZP (*P* = .49; MD = −1.75; 95%CI = −3.25 to 6.75) (Figure 3). In the general meta-analysis of a^∗^ values, the results showed a significant difference in the a^∗^ value between the nonaged and aged Y-TZP (*P* = .03; MD = −0.26; 95%CI = −0.51 to − 0.02), favoring the nonaged Y-TZP (Figure 4). In the general meta-analysis of b^∗^ values (Figure 5), no significant difference in the b^∗^ value between the nonaged and aged Y-TZP was found (*P* = .62; MD = 0.40; 95%CI = −1.17 to 1.97).

Subgroup analyses considering the steam autoclave duration (≤20 h vs. >20 h) were performed on the translucency and CIE L^∗^a^∗^b^∗^ coordinate data (Supplemental Figures 1, 2, 3, and 4). The results revealed that the steam autoclave duration contributed to the changes in the translucency and color of the aged Y-TZP ceramics (*P* all < .05). When the aging duration was ≤20 h, no significant differences in the TP, L^∗^, and b^∗^ values were found between aged and nonaged Y-TZP ceramics (*P* all > .05). However, when the aging duration was >20 h, significant differences in the TP, L^∗^, and b^∗^ values were found between the aged and nonaged Y-TZP ceramics (*P* all < .05). Significantly greater a^∗^ values were found in the aged Y-TZP ceramics than the nonaged ones, regardless of the aging duration. Furthermore, a subgroup analysis considering the type of monolithic translucent Y-TZP ceramic (3Y-TZP vs. 5Y-TZP) was performed on the translucency data (Supplemental Figure 5). No significant differences in the TP values were found between the subgroups (*P* = .45).

## 4. Discussion

To avoid the chipping of layered restorations, monolithic restorations have been promoted [1]. Monolithic restorations made of translucent Y-TZP ceramics, such as 3Y-TZP and 5Y-TZP, have become popular in recent decades. However, exposing Y-TZP ceramics directly to the oral environment may make them more susceptible to aging [16]. Therefore, this systematic review and meta-analysis was conducted to evaluate the effects of aging on the optical properties of monolithic translucent Y-TZP ceramics and can be considered the first in this study area.

In general, the esthetic outcome of monolithic Y-TZP restorations is mostly dependent on optical properties, including color and translucency. Translucency can be described as the quality of light passing through a material; this aspect is essential to the esthetic performance of dental restorations, which is crucial when selecting a restorative material [27]. The material brand, thickness, and composition (e.g., the yttrium content) have been reported to influence the optical properties of Y-TZP ceramics [56]. Other influencing factors may include the type and quantity of additives, the color shade, the coloring protocol (e.g., precolored or colored by immersion in coloring liquids), the presence of *c* phase content, the sintering temperature and time, and the surface roughness [34–36].

The TP and contrast ratio (CR) have been widely used to describe the translucency of dental materials [27]. Although the CR values were not considered in the present study due to insufficient data, the TP values have been confirmed to highly correlate with the CR values, and they can be used interchangeably [25–27]. The TP values of Y-TZP ceramics remained stable when the duration of hydrothermal aging was ≤20 h. However, after hydrothermal aging for >20 h (for the included studies, 40 to 100 h), the mean *Δ*TP value was 5.05, indicating that the Y-TZP ceramics had become significantly more opaque. Liu et al. [57] concluded that a CR difference (*Δ*CR) of 0.07 is the human perception threshold for translucency. Based on the correlation established between the TP and CR values, a *Δ*CR of 0.07 could be transformed into a *Δ*TP value of 2 [58]. Therefore, the translucency changes due to aging detected in the present study might be perceived by visual assessments. The change in translucency during aging is probably associated with the transformation of zirconia from the *t* phase to the *m* phase [40]. An increase in the *m* phase content due to aging causes the formation of microcracks and increases the surface porosity, therefore increasing the surface roughness, light scattering, and reflection [11–13]. The coexistence of the *t* and *m* phases after aging may also contribute to an increase in the difference in the refractive indices for an incident light beam, thereby decreasing the translucency [35, 59, 60]. The longer the aging duration is, the greater the *t*-to-*m* phase transformation (greater *m* phase content). An increase in the *m* phase content has been proposed to lead to increased opacity due to the abovementioned reasons [16, 61].

The color difference (Δ*E*) between 2 subjects can be calculated and used to report relative color changes. In dentistry, a Δ*E* of 2.7 is considered the threshold for a clinically unacceptable color difference according to ISO/TR 28642:2016 [44]. Apart from Δ*E* values, the National Bureau of Standards (NBS) units (NBS units = Δ*E* × 0.92) are also regarded as a means of visual assessment [62]. Significant differences in the a^∗^ values between the aged and nonaged Y-TZP ceramics were found, indicating that the Y-TZP ceramics appeared more reddish (greater a^∗^) after aging. According to the meta-analysis, the mean *Δ*L^∗^, *Δ*a^∗^, and *Δ*b^∗^ values were -1.75, -0.26, and 0.40, respectively. Based on the equation $∆E=\sqrt{{\left(\Delta L\right)}^{2}+{\left(\Delta a\right)}^{2}+{\left(\Delta b\right)}^{2}}$ [63], Δ*E* = 1.81, and NBS unit = 1.67, indicating that the color changes caused by aging might be noticeable. Similar to the TP values, the hydrothermal aging duration contributed to the changes in the CIE L^∗^, a^∗^, and b^∗^ values. The color (CIE L^∗^, a^∗^, and b^∗^ values) of the aged monolithic translucent zirconia remained unchanged when the aging duration ≤20 h. When the Y-TZP ceramics were aged for more than 20 h (for the included studies, 40 to 100 h), the Δ*E* = 5.73, indicating that the color changes were clinically unacceptable. Theoretically, thermal conditions may have an effect on the coloring pigments added to Y-TZP ceramics, causing pigment breakdown and resulting in color instability [64]. For example, some of the monolithic zirconia consists of minute amount of Fe_2_O_3_ as the pigment [65]. Fe_2_O_3_ has at least three isomorphs (*α*, *γ*, and *ε*) whereas they have different observable band gaps and oxygen valencies, such that *α* and *γ* are easily interchanged with each other even at room temperature [66]. Nevertheless, the exact mechanism of color instability is not clear and requires further investigation. In addition, the breakdown of colorants could also affect TP which was shown to be related to the changes in lightness and yellow–blue coordinates [67]. Thus, color is an important perceptive factor in the determination of the TP, given that TP is determined by the colorimeter/spectrophotometer and the thickness of the specimen tested. In other words, the experimental operating condition is critical and should receive more attention in the literature [27, 50].

Although hydrothermal aging is the most common method of accelerated aging, other less aggressive aging methods, such as thermocycling and exposure to ultraviolet light and water spray in a weathering machine, were used in the literature [23, 38]. Compared with steam autoclave, less aggressive aging methods, such as thermocycling and exposure to ultraviolet light and water spray in a weathering machine, presented less pronounced effects on the optical properties of Y-TZP ceramics. Dikicier et al. [23] reported that aging in a weathering machine for 300 h is equivalent to 1 year of clinical service. After 200 h of aging in a weathering machine, the Y-TZP ceramic presented only a minor color change, with a mean Δ*E* value of 1.19. Papageorgiou-Kyrana et al. [38] concluded that monolithic Y-TZP, either precolored or colored by immersion in staining solutions, can be considered color stable after 5000 thermocycles.

Although sensitivity analyses were conducted, no particular studies were responsible for generating heterogeneity. The high heterogeneity observed in the analyses could be explained by the various brands of test materials and the aging protocols, which may have led to a large change in the aging behavior. The present study was considered to have the following limitations: no subgroup analyses considering the type of Y-TZP ceramic on CIE L^∗^a^∗^b^∗^ coordinate data were performed because of insufficient data. Although the risk of bias assessment was based on previous studies [46, 47], the assessment methods may be improved by considering the topic of bias. Moreover, no clinical studies in this field were found; thus, there is weak evidence to support clinical recommendations.

Based on the present findings, the optical properties of monolithic translucent Y-TZP ceramics seemed to be stable after hydrothermal aging at 134°C and 0.2 MPa for ≤20 h. Clinically unacceptable changes in the translucency and color of monolithic translucent Y-TZP ceramics were found after hydrothermal aging for >20 h. The general consensus is that 1 h of autoclave aging is equivalent to 3 to 4 years *in vivo* [15], although a recent study reported that aging at 134°C and 0.2 MPa for 5 h was considered equivalent to 2 years of aging *in vivo* [24]. However, it is important to emphasize that in vivo data are needed to correlate the data from laboratory simulations and clinical situations. Therefore, further clinical studies are needed to clarify this hypothesis.

## 5. Conclusions

Within the limitations of this study, the following conclusions may be drawn:

1. The translucency and color of monolithic translucent Y-TZP ceramics remained stable when the duration of hydrothermal aging was less than 20 h.
2. Clinically unacceptable changes in the translucency and color of monolithic translucent Y-TZP ceramics were found when the duration of hydrothermal aging was more than 20 h.

## Figures and Tables

**Table 1:** Characteristics of included studies.

Author	Publication year	Generation of Y-TZP tested	Material tested	Aging protocol	Color measurements	Translucency measurements
Fathy et al.	2015	2^nd^ generation	3Y-TZP: Prettau (Zirkonzahn GmbH)	134°C and 0.2 MPa for 15 h	—	TP
Nakamura et al.	2016	2^nd^ generation	3Y-TZP: Incoris TZi (Dentsply Sirona)	134°C and 0.2 MPa for 5, 10, 20, and 40 h	—	TP and CR
Alghazzawi TF	2017	2^nd^ and 3^rd^ generations	3Y-TZP: Zenostar T (Ivoclar Vivadent), Zirlux (Henry Schein), Katana HT (Kuraray Noritake Dental), Bruxzir (Glidewell Laboratories), DD-BioZX [2] (Dental Direkt GmbH), NexxZr (Sagemax Bioceramics) 5Y-TZP: DD-cubeX^2^ (Dental Direkt GmbH)	134°C and 0.2 MPa for 20, 40, 60, 80, and 100 h	CIE L^∗^a^∗^b^∗^ coordinates and color difference (Δ*E*)	TP and CR
Putra et al.	2017	2^nd^ and 3^rd^ generations	3Y-TZP: Lava Plus (3M ESPE) 5Y-TZP: Bruxzir Anterior (Glidewell Laboratories), Katana UT (Kuraray Noritake Dental)	134°C and 0.2 MPa for 5, 50, and 100 h	—	Percentage of total transmittance of light (Tt%)
Rafeal et al.	2018	2^nd^ generation	3Y-TZP: Prettau (Zirkonzahn GmbH)	134°C and 0.3 MPa for 1 h	Color differences (Δ*E*_00_)	—
Kim and Kim	2019	2^nd^ generation	3Y-TZP: Katana ML (Kuraray Noritake Dental)	134°C and 0.2 MPa for 1, 3, 5, and 10 h	CIE L^∗^a^∗^b^∗^ coordinates and color differences (Δ*E*_00_)	TP
Walczak et al.	2019	2^nd^ generation	3Y-TZP: Cercon ht (Degudent GmbH), Bruxzir (Glidewell Laboratories), Zenostar T (Ivoclar Vivadent), Lava Plus (3M ESPE)	134°C and 0.2 MPa for 5 h	—	TP and CR
Kou et al.	2019	3^rd^ generation	5Y-TZP: DD-cubeX^2^ (Dental Direkt GmbH), Bruxzir Anterior (Glidewell Laboratories)	134°C and 0.2 MPa for 10 h	—	Visible transmittance
Shen et al.	2020	2^nd^ and 3^rd^ generations	3Y-TZP: Lava Plus (3M ESPE) 5Y-TZP: Katana UTML (Kuraray Noritake Dental)	134°C and 0.2 MPa for 20 h	—	TP
Benalcazar Jalkh et al.	2020	2^nd^ generation	3Y-TZP: Zpex (Tosoh)	134°C and 0.2 MPa for 20 h	—	TP and CR
de Araújo-Júnior et al.	2020	2^nd^ generation	3Y-TZP: Zirconn translucent (VIPI)	134°C and 0.2 MPa for 20 h	Color difference (Δ*E*)	TP and CR
Cokic et al.	2020	2^nd^ and 3^rd^ generations	3Y-TZP: CEREC Zirconia medi S (Dentsply Sirona), Incoris TZi (Dentsply Sirona) 5Y-TZP: Katana STML (Kuraray Noritake Dental)	134°C and 0.2 MPa for 60 h	—	TP and CR
Lopes et al.	2020	2^nd^ generation	3Y-TZP: Zpex (Tosoh)	134°C and 0.2 MPa for 20 h	Color difference (△E)	TP and CR

TP: translucency parameter; CR: contrast ratio.

**Table 2:** Risk of bias in included studies.

Author	Publication year	Sample size calculation	Randomization	Aging protocol	Statistical analysis	Ceramic sintering	Blinded examiner	Risk of bias
Fathy et al.	2015	N	N	Y	Y	Y	N	Medium
Nakamura et al.	2016	N	N	Y	Y	Y	N	Medium
Alghazzawi TF	2017	N	N	Y	Y	Y	N	Medium
Putra et al.	2017	N	N	Y	Y	Y	N	Medium
Rafeal et al.	2018	N	N	Y	Y	N	N	High
Kim and Kim	2019	N	N	Y	Y	N	N	High
Walczak et al.	2019	N	N	Y	Y	Y	N	Medium
Kou et al.	2019	N	Y	Y	Y	Y	N	Low
Shen et al.	2020	N	N	Y	Y	Y	N	Medium
Benalcazar Jalkh et al.	2020	N	Y	Y	Y	Y	N	Low
de Araújo-Júnior et al.	2020	N	N	Y	Y	Y	N	Medium
Cokic et al.	2020	N	N	Y	Y	Y	N	Medium
Lopes et al.	2020	N	N	Y	Y	Y	N	Medium

## Data Availability

The data supporting the present results are included in this article.

## Supplementary Materials

Supplementary materials Supplemental Table 1: search terms and combinations used in the literature search. Supplemental Figure 1: forest plot summarizing TP values of aged and nonaged Y-TZP ceramics (subgroup: steam autoclave duration). CI: confidence interval; SD: standard deviation. Supplemental Figure 2: forest plot summarizing L^∗^ values of aged and nonaged Y-TZP ceramics (subgroup: steam autoclave duration). CI: confidence interval; SD: standard deviation. Supplemental Figure 3: forest plot summarizing a^∗^ values of aged and nonaged Y-TZP ceramics (subgroup: steam autoclave duration). CI: confidence interval; SD: standard deviation. Supplemental Figure 4: forest plot summarizing b^∗^ values of aged and nonaged Y-TZP ceramics (subgroup: steam autoclave duration). CI: confidence interval; SD: standard deviation. Supplemental Figure 5: forest plot summarizing TP values of aged and nonaged Y-TZP ceramics (subgroup: type of Y-TZP ceramic). CI: confidence interval; SD: standard deviation.
