# Supplementary material for: Effects of Aging on the Color and Translucency of Monolithic Translucent Y-TZP Ceramics: A Systematic Review and Meta-Analysis of In Vitro Studies
Source: Biomed Res Int. 2021 Jan 25;2021:8875023. doi: 10.1155/2021/8875023 (PMC7857873; doi:10.1155/2021/8875023)
Supplement: Supplementary materials — Supplemental Table 1: search terms and combinations used in the literature search. Supplemental Figure 1: forest plot summarizing TP values of aged and nonaged Y-TZP ceramics (subgroup: steam autoclave duration). CI: confidence interval; SD: standard deviation. Supplemental Figure 2: forest plot summarizing L∗ values of aged and nonaged Y-TZP ceramics (subgroup: steam autoclave duration). CI: confidence interval; SD: standard deviation. Supplemental Figure 3: forest plot summarizing a∗ values of aged and nonaged Y-TZP ceramics (subgroup: steam autoclave duration). CI: confidence interval; SD: standard deviation. Supplemental Figure 4: forest plot summarizing b∗ values of aged and nonaged Y-TZP ceramics (subgroup: steam autoclave duration). CI: confidence interval; SD: standard deviation. Supplemental Figure 5: forest plot summarizing TP values of aged and nonaged Y-TZP ceramics (subgroup: type of Y-TZP ceramic). CI: confidence interval; SD: standard deviation. [file 8875023.f1.docx]

| Database | Search terms and combinations used |
| --- | --- |
| Medline via Pubmed | (((zirconium [MeSH Terms] OR zirconia OR zirconium oxide OR Y-TZP OR yttria stabilized polycrystalline tetragonal zirconia OR yttria stabilized tetragonal zirconia)) AND (aging OR accelerated aging OR low temperature degradation OR hydrothermal aging)) AND (color OR translucency OR optical characteristic OR optical property) |
| Embase | (‘zirconium’/exp OR ‘zirconia’ OR ‘zirconium oxide’ OR ‘Y-TZP’ OR ‘yttria stabilized polycrystalline tetragonal zirconia’ OR ‘yttria stabilized tetragonal zirconia’) AND (‘aging’ OR ‘accelerated aging’ OR ‘low temperature degradation’ OR ‘hydrothermal aging’) AND (‘color’ OR ‘translucency’ OR ‘optical characteristic’ OR ‘optical property’) AND [embase]/lim |
| Web of Science | (“zirconium” OR “zirconia” OR “zirconium oxide” OR “Y-TZP” OR “yttria stabilized polycrystalline tetragonal zirconia” OR “yttria stabilized tetragonal zirconia”) AND (“aging” OR “accelerated aging” OR “low temperature degradation” OR “hydrothermal aging”) AND (“color” OR “translucency” OR “optical characteristic” OR “optical property”) |
| Cochrane Library | (“zirconium” OR “zirconia” OR “zirconium oxide” OR “Y-TZP” OR “yttria stabilized polycrystalline tetragonal zirconia” OR “yttria stabilized tetragonal zirconia”) AND (“aging” OR “accelerated aging” OR “low temperature degradation” OR “hydrothermal aging”) AND (“color” OR “translucency” OR “optical characteristic” OR “optical property”) |

**Appendix**

Supplemental Table 1 Search terms and combinations used in the literature search

Supplemental Figure 1. Forest plot summarizing TP values of aged and non-aged Y-TZP ceramics (subgroup: steam autoclave duration).


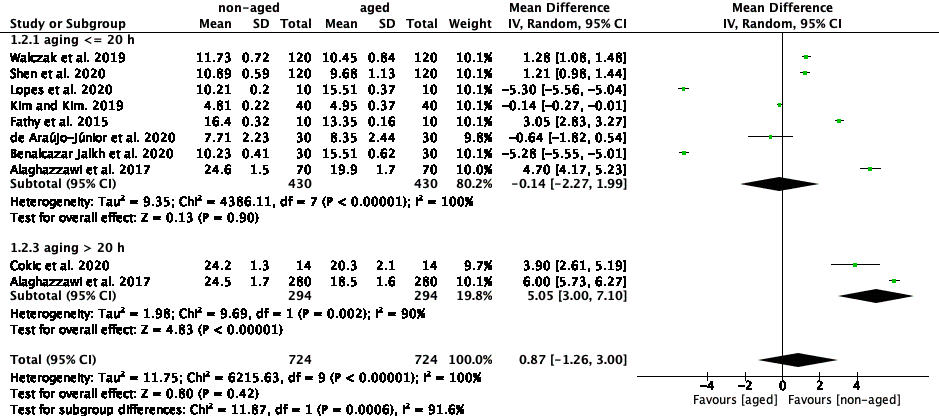


CI, confidence interval; SD, standard deviation.

Supplemental Figure 2. Forest plot summarizing L* values of aged and non-aged Y-TZP ceramics (subgroup: steam autoclave duration).


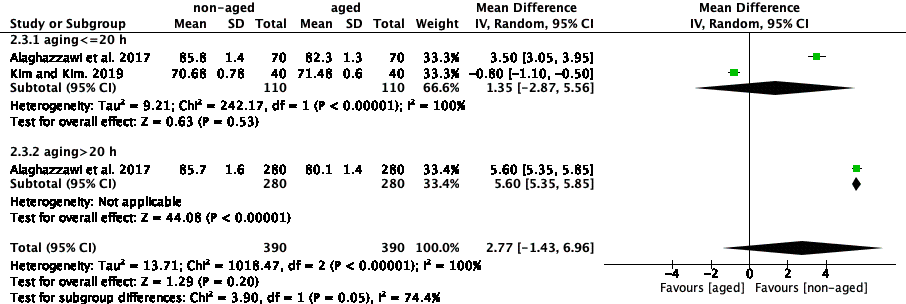


CI, confidence interval; SD, standard deviation.

Supplemental Figure 3. Forest plot summarizing a* values of aged and non-aged Y-TZP ceramics (subgroup: steam autoclave duration).


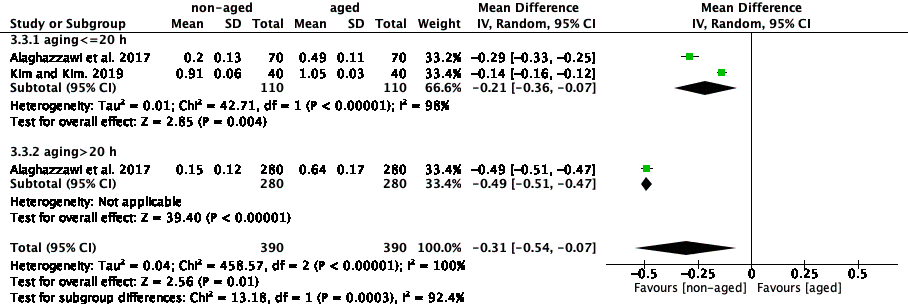


CI, confidence interval; SD, standard deviation.

Supplemental Figure 4. Forest plot summarizing b* values of aged and non-aged Y-TZP ceramics (subgroup: steam autoclave duration).


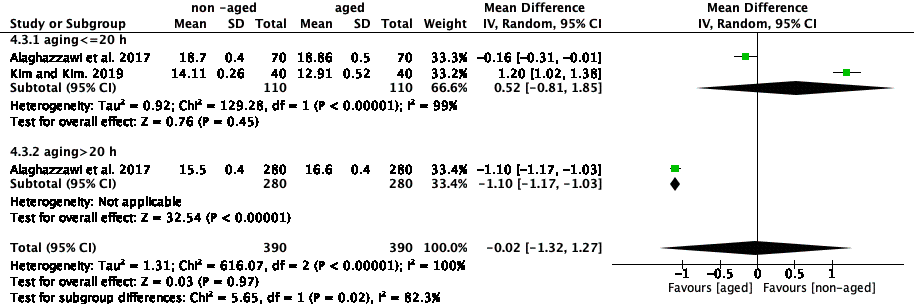


CI, confidence interval; SD, standard deviation.

Supplemental Figure 5. Forest plot summarizing TP values of aged and non-aged Y-TZP ceramics (subgroup: type of Y-TZP ceramic).


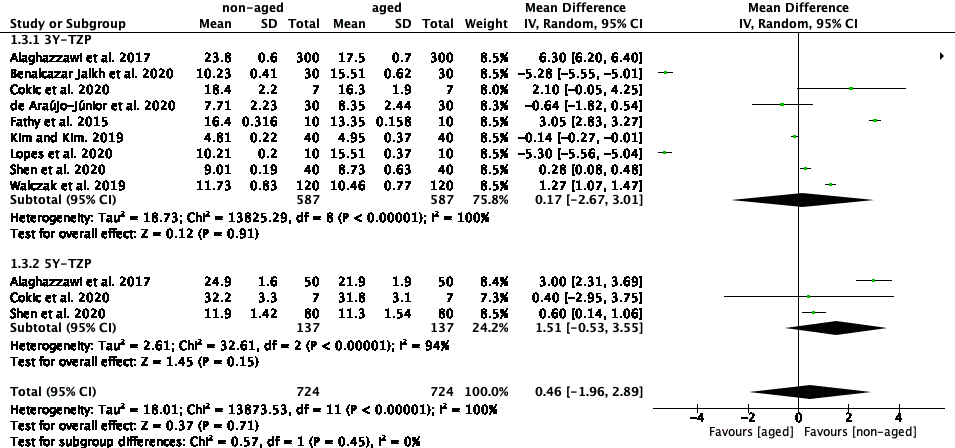


CI, confidence interval; SD, standard deviation.
